# Data set about local mapping of the Earth's magnetic field

**DOI:** 10.1016/j.dib.2018.01.112

**Published:** 2018-02-08

**Authors:** Daniel Polčin

**Affiliations:** Department of Informatics, Faculty of Education, Catholic University in Ruzomberok, Hrabovská 1, 034 01 Ružomberok, Slovakia

**Keywords:** Magnetic field, Magnetic induction, Measurement, Measuring instruments, Faraday's law, Monitoring

## Abstract

The data presented in this article are related to the research article entitled "Magnetic field and its experimental measurement in teaching in high schools" (J. Beňuška, D. Polčin, 2016) [1].

The article describes the possibilities of relatively accurate experimental measurement of magnetic field in any geographical conditions, in both exteriors and interiors, without the need for special instrumentation, with minimal financial costs.

The data set is publicly available to allow the comparison of magnetic induction values in given latitudes with the other latitudes by the given methods and a critical analysis of the applicability of these methods in areas and locations without more demanding instrumentation.

**Specifications Table**

**Table:** 

Subject area	Geophysics, Applied Geophysics
More specific subject area	Local and regional geomagnetic mapping for a more accurate description of dynamic magnetic field changes
Type of data	Tables, charts, text file
How data was acquired	1 Measurement of the horizontal component of the magnetic induction of the Earth's magnetic field by a thread rotation
	2 Measurement of the horizontal component of the magnetic induction of the geomagnetic field, using a circular coil
Data format	Raw, analysed
Experimental factors	The magnitude of the magnetic field induction of the Earth's magnetic field was measured by two simple methods to compare their accuracy.
Experimental features	The regional dynamics of magnetic field changes were monitored by comparing of measurements in different time periods.
	There were regional dynamics of the Earth's magnetic field changes observed in the given area.
Data source location	Ružomberok, Slovakia, 49°04'57.9"N; 19°17'36.0"E
Data accessibility	The data are available with this article.

**Value of the data**

• The data provide information about the horizontal magnetic induction component of the Earth's magnetic field in the region and may be used by other scientists at geomagnetic mapping.
• The observed dynamics of local magnetic field changes can supplement the data on its spatial and temporal dynamic properties for specialized observatories.
• These data allow other researchers to complement the network of geomagnetic observations and thus partly specify the magnetic field distribution map

## 1 Data

**Fig. 1:**
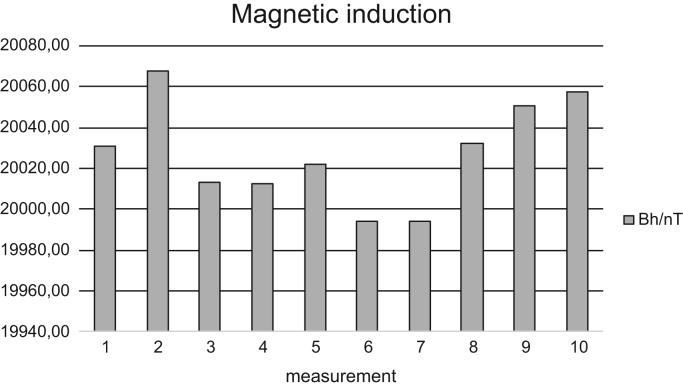
Measured values of horizontal component of magnetic induction B_h_ of the Earth's magnetic field by a thread rotation - Dec 18, 2015.

**Fig. 2:**
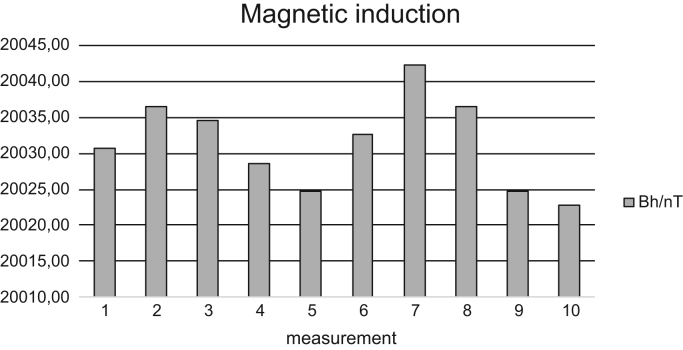
Measured values of horizontal component of magnetic induction Bh of the Earth's magnetic field by a circular coil - Dec 18, 2015.

**Fig. 3:**
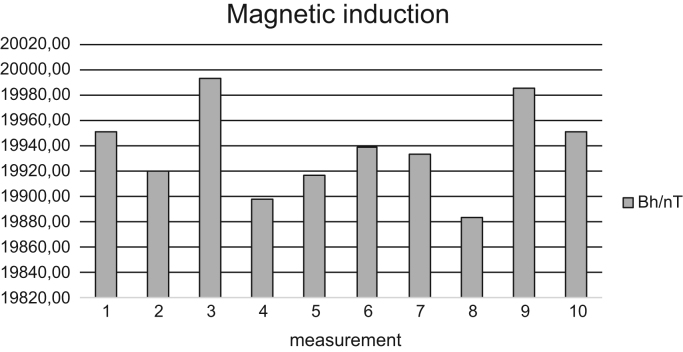
Measured values of horizontal component of magnetic induction Bh of the Earth's magnetic field by a thread rotation - Jun 24, 2016.

**Fig. 4:**
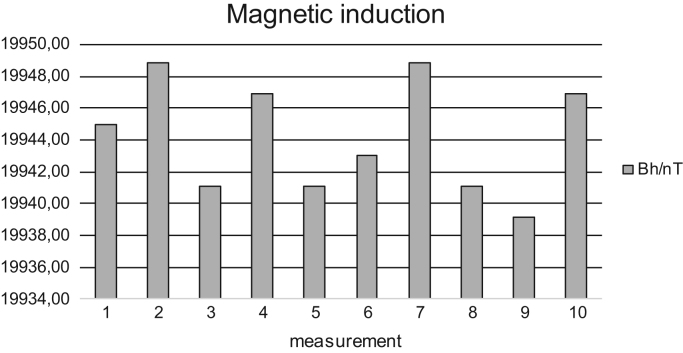
Measured values of horizontal component of magnetic induction Bh of the Earth's magnetic field by a circular coil - Jun 24, 2016.

The data set of this article provides information about the measured magnitude of the horizontal magnetic induction component of the Earth's magnetic field in the selected geographical location. Fig. 1, Fig. 2, Fig. 3, Fig. 4 with corresponding Table 1, Table 2, Table 3, Table 4 show the measured values in two time periods - December 18, 2015 and June 24, 2016.

**Table 1:** Measured values of horizontal component of magnetic induction B_h_ of the Earth's magnetic field by a thread rotation - Dec 18, 2015 (The triangle surface: S = 4.07 m^2^).

n	10*T*/s	*T* /s	*U*/mV	B_h_/nT	
1	10.87	1.087	0.30	20030.71	$B_{h}=\frac{UT}{4S}$
2	10.89	1.089	0.30	20067.57	
3	10.51	1.051	0.31	20012.90	
4	10.86	1.086	0.30	20012.29	
5	11.24	1.124	0.29	20022.11	
6	10.85	1.085	0.30	19993.86	
7	10.50	1.050	0.31	19993.86	
8	10.52	1.052	0.31	20031.94	
9	10.53	1.053	0.31	20050.98	
10	11.26	1.126	0.29	20057.74	
Average				20027.40

**Table 2:** Measured values of horizontal component of magnetic induction Bh of the Earth's magnetic field by the circular coil - Dec 18, 2015.

n	N	d/m	I/mA	B_h_/nT	
1	31	0.2	102.89	20030.63	$B_{h}=\mu \frac{NI}{d}$
2	31	0.2	102.92	20036.47	
3	31	0.2	102.91	20034.52	
4	31	0.2	102.88	20028.68	
5	31	0.2	102.86	20024.78	
6	31	0.2	102.90	20032.57	
7	31	0.2	102.95	20042.31	
8	31	0.2	102.92	20036.47	
9	31	0.2	102.86	20024.78	
10	31	0.2	102.85	20022.84	
Average				20031.40	

N circular coil threads number.

I current passing through coil threads

d coil diameter

μ environment permeability (for air approximately as vacuum 4.3,14.10^−7^ N.A^−2^).

**Table 3:** Measured values of horizontal component of magnetic induction Bh of the Earth's magnetic field by a thread rotation - Jun 24, 2016 (The triangle surface: S = 4.07 m^2^).

n	10*T*/s	*T* /s	*U*/mV	B_h_/nT	
1	11.20	1.120	0.29	19950.86	$B_{h}=\frac{UT}{4S}$
2	10.81	1.081	0.30	19920.15	
3	10.85	1.085	0.30	19993.86	
4	11.17	1.117	0.29	19897.42	
5	11.58	1.158	0.28	19916.46	
6	10.82	1.082	0.30	19938.57	
7	11.59	1.159	0.28	19933.66	
8	10.79	1.079	0.30	19883.29	
9	11.62	1.162	0.28	19985.26	
10	11.60	1.160	0.28	19950.86	
Average				19937.04

**Table 4:** Measured values of horizontal component of magnetic induction B_h_ of the Earth's magnetic field by the circular coil - Jun 24, 2016.

n	N	d/m	I/mA	B_h_/nT	
1	31	0.2	102.45	19944.97	$B_{h}=\mu \frac{NI}{d}$
2	31	0.2	102.47	19948.86	
3	31	0.2	102.43	19941.07	
4	31	0.2	102.46	19946.91	
5	31	0.2	102.43	19941.07	
6	31	0.2	102.44	19943.02	
7	31	0.2	102.47	19948.86	
8	31	0.2	102.43	19941.07	
9	31	0.2	102.42	19939.13	
10	31	0.2	102.46	19946.91	
			Average	19944.19	

N circular coil threads number

I current passing through coil threads

d coil diameter

μ environment permeability (for air approximately as vacuum 4.3,14.10^−7^ N.A^−2^)

## 2 Experimental design, materials and methods

### 2.1 Measurement of the horizontal component of the magnetic induction of the Earth's magnetic field by a thread rotation

The experiment is designed to measure the magnitude of the magnetic induction of the Earth's magnetic field in our latitude by means of a rotating conductor in which the voltage is induced by Faraday's law of electromagnetic induction, with changes in magnetic induction current through the surface described by the conductor.

Materials:

fixed wire (20 m) loaded in the middle by a tennis ball, milivoltmeter, stopwatch, length gauge, compass to determine the correct direction - the surface plane of the thread must be perpendicular to the direction south-north.

When spinning the wire in the magnetic field of the Earth in the south-north direction, there is a constant change in the thread area through which the magnetic induction lines pass. This changes the magnetic induction flux and induces the voltage we measured in the thread. The magnetic induction value was calculated from it.

### 2.2 Measurement of the horizontal component of the magnetic induction of the geomagnetic field, using a circular coil

The principle consists in comparing the magnitude of the magnetic field induced by the electric current passing through the coil and the magnitude of the horizontal component of the Earth's magnetic field.

Materials:

$$\begin{array}{c} \mathrm{circular}\;\mathrm{coil}\;\left(31\;\text{threads},\;\mathrm{diameter}\;20\;\mathrm{cm}\right),\;\mathrm{paper}\;\text{box},\;\text{compass},\\ \text{Ue}=4.5\;V\;\mathrm{voltage}\;\mathrm{source}\;\left(\mathrm{battery}\right),\;100\;\Omega \;\text{potentiometer},\;\mathrm{ampermeter}\;\left(\mathrm{up}\;\mathrm{to}\;300\;\mathrm{mA}\right). \end{array}$$

By setting the electric current in a circular coil so that the magnet against the north is at an angle of 45 °, we get the equation of magnetic induction magnitude of the magnetic field of the coil B_C_ and of the Earth B_Z_. From this equation, magnetic induction values were calculated.

Finally, we compared the data measured with both methods. We analyzed the dynamics of magnetic field changes by comparing data acquired in two different time periods.
